# Reviews and Book Notices

**Published:** 1881-09

**Authors:** 


### Reviews and Book Notices.

ARTICLE X.—A PRACTICAL TREATISE ON DISEASES OF THE SKIN. By Louis A. Duhring, M.D., Professor of Diseases of the Skin in the Hospital of the University of Pennsylvania, etc. Second edition, revised and enlarged. 8vo, pp. 644. Philadelphia: J. B. Lippincott & Co., 1881.

The revised and enlarged second edition of this work, whose previous issue greatly commended itself to the profession, appears with emendations and additions which largely enhance its practical value. The too prevalent fashion of preparing second editions of a work for the press, has here evidently found no favor. Those who have had reason to know the conscientious and scrupulous care with which the author has devoted himself to the other labor he has set before him, will be prepared to find in these pages a further proof of the excellent quality of his work.

Respecting the more frequently occurring diseases of the skin, the special excellence of this volume has long been recognized in the accuracy of its definitions, in the simplicity and fidelity of its clinical histories, in the absence of untenable theories and of an intolerably redundant nomenclature, and in the value of the therapeutic measures recommended both with a view to general and topical treatment. Naturally, in none of these particulars does the new edition fall short of the excellence of its predecessor. We look over its leaves and find them laden with the fruit of ripe experience—a wise discrimination between observation and theory, and a comprehensive familiarity with the literature of each subject presented.

As regards the diseases of less frequent occurrence, those which more commonly fall under the observation of the expert, we find that the chapters on dysidrosis, and pompholix, hæmatidrosis, scleroderma, morphœa, atrophia cutis, hypertrophy of the hair, atrophy of the hair, scrofuloderma, syphiloderma, and carcinoma have been those chiefly enlarged by important additions; and it is worthy of note that on those subjects, the contributions of the author to current literature have been in many cases of the greatest value.

The new articles all embrace subjects of special interest to Americans, as the rare diseases to which the attention of the reader is called are yearly of more frequent observation here, probably not because they are of increasing frequency, but because the labors of American dermatologists have directed such attention to them that they are more frequently recognized. It was said of Thoreau, by Mr. Emerson, that he expected one day to see the *Victoria regia* growing in the vicinity of Concord, and certainly the number of cutaneous diseases recognized abroad and hitherto supposed to be of the greatest infrequency in America, is rapidly diminishing. The new descriptions found here, relate to uridrosis, phosphorescent sweat, urticaria pigmentosa, dermatitis circumscripta herpetiformis, pityriasis maculata et circinata, dermatitis exfoliativa, dermatitis papillaris medicamentosa, dermatitis gangrænosa, dermatitis capillitii, fungoid neoplasmata, tuberculosis cutis, podelcoma, ainhum, perforating ulcer of the foot, and myoma cutis.

Doubtless there are those who will glance through a list of these names and be conscious of a sensation of dismay. The terms are many of them unfamiliar to the fairly-well educated physician. The subjects to which they refer are to him of small practical moment, he may conclude. Possibly he will dismiss the whole with a too common reflection, that dermatology is wedded to its cumbrous nomenclature and mysterious refinements above the level of the average intelligence. It is worth while to consider this reproach for a moment.

For it is a reproach which has not lacked public expression. The annual meetings of the American Dermatological Association have been criticised in our medical journals, in consequence of the time thus spent in discussion of rare forms of cutaneous disease; and it is sufficiently common to hear medical men of position and education admit their ignorance of the general subject of skin diseases, attributing this to the large number of rare forms of such maladies and the confusion existing with respect to their designation.

Now, without stopping to charge these sins of nomenclature upon all other departments of scientific labor, we cannot fail to remember that, especially of late years, the terms employed in chemistry and perhaps more particularly in connection with new operations upon the female genitals, have been of the most complicated and perplexing character; is is, however, needful to note that the newer terms employed by the dermatologist are those connected with distinct pathological processes or the objective evidences of such processes, while the others to which we have referred are more generally the terms demanded by the refinements of art, or by the requirements of exactness in technical expression. To know the rule in dermatology, one must know the exceptions; to be able to pronounce with precision upon the most common of diseases, one must be positive in the exclusion of every other. The exceptions, though rare, are visible alike to physician and patient, and clamorously assert their identity in their behavior. They betray their differences also in public, and to the eye of the individual who is not consulted as to their nature. Again, it is always necessary to get rid of the doctrines respecting ghosts, before one is in position to discuss a fact of nature. To establish the fact of a new or rare cutaneous disease, one is placed under the necessity of clearing away an incredible quantity of rubbish.

Look for a moment at the title given here "Dermatitis Exfoliativa." The other names mentioned by our author under this head, as employed by writers, are, "General Exfoliative Dermatitis," "Recurring Exfoliative Dermatitis," "Desquamative Scarlatiniform Erythema," "Recurrent Acute Eczema," "Acute General Dermatitis," "Recurrent Exfoliative Erythema," and "Scarlatiniform Exfoliative Dermatitis." Here unquestionably there is need of a single term, by which may be understood the varieties of exanthem described by such writers as Baxter, Féréol, Fagge, Pye-Smith, and, among the Americans, by such writers as Bulkley and G. H. Fox. Our author might have added here, the names also of Guibout, of Paris, and Jamieson, of Edinburgh, had the recent papers of these last-named gentlemen appeared before the book went to press. Surely this is a subject requiring attention in a treatise devoted to the consideration of cutaneous disease.

Here, again, we find the evidences of our author's conservatism. He restricts the use of the term we have given, to certain unusual and occasionally grave forms of disease, characterized by an acute erythematous, more rarely vesicular or bullous, inflammation, localized or generalized, accompanied by a more or less marked febrile disturbance, accompanied or followed by varying degrees of desquamation or exfoliation of the epidermis, and marked by a tendency to relapse. The disorder he thus describes is to be distinguished from the recognized varieties of eczema and psoriasis, and from pityriasis rubra and pemphigus foliaceus. But how are these distinctions to be established, especially in face of the fact, that "much diversity of opinion exists as to the true nature of the cases * * * and it is difficult to determine from the reports whether they illustrate the same process or different diseases." Difficult, indeed, it is; one is strongly tempted to disregard alike authorities and minor differences of fact under the circumstances, and to agree with Jamieson that the term "general exfoliative dermatitis" should be made to include the very diseases from which we are here told that the conditions included by that term should be carefully differentiated.

Now it is in just such periods, when our knowledge of the different phenomena displayed under different circumstances by a single pathological condition is widening and deepening, that a wise conservatism is requisite. True, there is a wide distinction between typical cases of pemphigus foliaceus, for example, where the dried contents of a collapsed bleb furnish the material of the papery flakes cast off from the surface, and that disorder characterized merely by "bullous inflammation followed by various degrees of desquamation of the epidermis with a tendency to relapse." Yet the evidences of the existence of intermediate forms between these extremes are not wanting, and Jamieson (*Edinburgh Med. Journ.*, April, 1880, p. 879) cites several cases which it would be exceedingly difficult to assign exclusively to the one category or the other. One such has also been reported by ourselves (CHICAGO MED. JOURN. AND EXAM., February, 1881), under the title Pityriasis Rubra, where there was coexistence of very extensive epidermal desquamation, with febrile accesses of fatal termination, and where the surface exhibited marble-sized projections, which were found, post-mortem, to contain a creamy pus, and which served to give a particular character to the malady. We are not yet ready for a distinct recognition of all these rare and severe cutaneous manifestations as members of a single family, and yet, if the signs do not fail, to that complexion we shall eventually come. Our author has wisely given us a provisional recognition of these typical states, under the title "Dermatitis Exfoliativa."

We could in this way go over all the interesting ground traversed here for the first time, by one well qualified to pronounce with decision at every step, but we must be content by reminding those who find it necessary to consult a treatise of this sort, of the importance to themselves of possessing a brief yet precise description of each of the rarer cutaneous diseases, especially when it is requisite to come to a diagnosis by the processes of exclusion. It is for these reasons that we take especial pleasure in commending Dr. Duhring's book to the practitioner as well as to the student of medicine, and it is probably true that the marked success of the first edition was based upon its value in just this particular. Of it can be said now, even more confidently than on its first appearance, that it is the best treatise in the English language on the subject of diseases of the skin. As a text-book, it stands without a rival, and as a monument to the author's painstaking and indefatigable industry, it is one upon which he can well be congratulated. It is the production of books like this, which has brought to the profession in this country a degree of recognition and respect abroad which we could not in any other way have attained, and it is this which has held up to our own countrymen a standard of literary excellence in authorship which has proved of inestimable value.

The new illustrations in the chapter on anatomy of the skin are very creditable drawings by Dr. Van Harlingen, and serve well to exhibit the relations between the protoplasmic bodies in the mucous layer, formerly described as hanging together by spines or prickles at their points, and clearly shown by Heitzmann to be generally connected by a thread-like network of living matter, traversing the lifeless cement substance between the epithelia.

The typography of the book is beyond criticism, and in other respects the publishers have done their part in its production with a most commendable success.—*American Journal of the Medical Sciences.* J. N. H.

ARTICLE XI.—LECTURES ON DISEASES OF THE NERVOUS SYSTEM, ESPECIALLY IN WOMEN. By S. Weir Mitchell, M.D., Member of the National Academy of Sciences (and of ten medical societies), etc. With five plates. 12mo, cloth, pp. 238. $1.75. Philadelphia: Henry C. Lea's Son & Co., 1881. Chicago: Jansen, McClurg & Co.

This is a very ill chosen title for a contribution to the study of some phenomena related to hysteria, chorea, and epilepsy. The five lithographic plates are five tables exhibiting the barometric pressure, atmospheric temperature, and other conditions of the weather in their relation to chorea and infantile palsy. They are of doubtful value. This monograph is pleasantly written, contains not one technical term, treats of some of the most common events of life; all this with little pretension to scientific researches, and will find its sphere of usefulness as a means of advertising among nervous people, at least this idea will suggest itself to many readers.

The author reports some very interesting cases that came to him from Indiana, Kentucky, Ohio, Mississippi, Canada, Maryland, Rhode Island, Massachusetts, New Jersey, Connecticut, etc., and he cured several of them. One must not judge from this however, that this production of a specialist, and "common sense doctor" is devoid of merits. It treats of some subjects never before so keenly investigated as by Dr. Mitchell, whose manner of treatment is highly commendable and too often overlooked by the "busy practitioner." No doubt it will benefit the latter to peruse in an abstract the substance of these lectures, since he will not have time to read such prolix literature. The author's words are preserved as much as possible, and so also the order of chapters.

### PARALYSES OF HYSTERIA.

*Causation.*—It is most common among the ill-fed, needy, and worried; people that have lost money, and those who lack distinct purposes and aims in life. The palsy may be partly real, partly pure weakness, partly loss of power from want of belief in being able to move. In practice it is usual to find two or three causes acting to assist one another. Four cases of hemiplegia on the left were met to one on the right, and the face was not usually involved. All the senses are liable to be blunted. Profound emotions act as a cause.

*Symptoms.*—You will see women with a low, whining, bleating voice that is by itself a tell-tale of the kind of will-less ataxia which seems to cripple the mind no less than the body. These are the hard cases to relieve. In fact, even with the best of self help from the patient, the cure of any one of these cases is a long and arduous course of education. All hysteria is not after a time within control of the sick person, and no human sagacity will suffice to enable us to predict results from profound emotions on the patient.

*Treatment.*—The cures of these cases are to be made by a slow, steady, hopeful training of the will powers through every-day effort, which needs some caution not to err in the way of excess. There is no short cut; no royal road.

### HYSTERICAL PARESES.

In some cases of this, nerve conduction is retarded, and gives rise to such incoördinate action as they exhibit. This is made worse by excluding the guiding influence of vision. It is limited for the most part to the more complex movements. This is common in grave hysteria.

*Course.*—The history of hysteria is sometimes one of years, and in certain cases, either at the outset or after more or less of the strange drama of this disease has been played, the patient falls into a state of inertness of mind and body, which I am forced, for lack of a better name, to call hysteric paresis. This is one of those disorders which adds many recruits to that large class which some one has called "bed cases," and which are, above all things, distinguished by their desire to remain at rest. They are liable to have relapses.

*Treatment.*—In grave cases it is best to teach the patient to creep, which is an easy and natural mode of training for the walk. The progress to creeping is easy, then comes the lesson of kneeling and pushing a chair; and last, that of standing in a corner or by a chair. No drug has a direct value. An occasional benefit is derived from induction currents. What you have to do is to rectify with care positive uterine troubles, to treat defects of nutrition, to relieve the anæmia so apt to exist in hysteria, to see that every function is well cared for; and last, not least, to learn what need there is to alter the moral surroundings of your patient, and then with kind and patient care, and an unbinding will, to bring about the changes she may seem to require.

MIMICRY OF DISEASE.

*Etiology.*—The elements out of which these disorders arise are deeply human, and exist in all of us in varying amount, while many of the determining and conditioning factors come from accidental, or, at least, external agencies. The hysterical state, the nervous temperament, general nervousness, neurasthenia. In the over-sensitive, emotional and timid. It is with some people a morbid birth-gift, with some an inheritance, and in its worst shapes it is made or acquired by misuse of alcohol or tobacco, or tea or coffee. It comes from failing health. Such a state is usually slowly created; but a permanent state of general nervousness may be evolved by the accident of a moment, when precedent conditions favor it. Then every one has some capacity for mentally influencing or disturbing functions of the body which usually are not under the control of volition. In some hysteric or nervous states this power becomes enormously increased. There exists in all of us, feebler in age and more potent in childhood, a tendency to automatic and unconscious imitation which is the parent of a good deal of the mimicry of disease. In this disease, indeed, we find women, and men too, passing into a mental state in which they are really much like people in dreams. Their power to reason on the phenomena of the senses leaves them, and what they conceive to be the case takes the place of that which is. This is to be met with, to some extent, in all grave hysteric cases. In these cases the emotions are no more under control than in a dream, and the pains are little. A case recovered after a few days, but sometimes the return to health and healthy ideas exacts a long and tiresome trouble. Some children enjoy the important rôle of being ill.

*Treatment.*—The need for care in discussing symptoms before nervous women or children, the necessity of early apprehension of the true state of things in simulated disease, and the wisdom of acting decisively when once we are sure of our ground are obvious.

Unusual Forms of Spasmodic Affections in Women.—The woman who habitually has hysterical spasms has something wrong with her general health. She is anæmic, or has lost general tone; there is nervous exhaustibility. If we can relieve it we cure the convulsions.

Tremor, Chronic Spasms.—Tremor is an expression of weakness in old age, fatigue, convalescence, organic diseases, or toxic states, transient emotions, fear, excitement, anger, or grief; disappointed love, abuse of alcohol, etc. It is most persistent as a result of nervous wounds. There is no abrupt recovery. The treatment largely consists in experimenting with these cases. Hypodermic injection of atropia locally in nervous spasms is useful. So is massage, extension by splints and apparatuses.

Chorea of Childhood.—Stormy weather, spring time and schools influence the disease, which has a tendency to recur. Puberty is causative also. This is essentially a disease of cities, and is rare in the Negro. Arsenic is the best remedy.

Habit Chorea—also a disease of childhood—consists in rapid and repeated twitching of some muscles; rapid winking, shrugging of the shoulder, etc. Most common in girls from seven to fourteen years of age. There has been some fall from the plane of health. A cause cannot always be detected. The child's restraining power is not well exercised. Good and careful diet, light gymnastics, no school, gentle aperients, full doses of arsenic, form the best treatment. Hypodermic injections of Fowler's solution without the lavender, thrice a week, in doses rising from two to twelve drops, is the best method of administration.

Disorders of Sleep in Nervous or Hysterical Persons.—This corresponds as much to epilepsy as habit chorea does to chorea. Self control, and some fatigue will relieve it.

Vaso-motor and Respiratory Disorders in the Nervous or Hysterical.—The *pulse* is apt to be as rapid as 90, 100, 130 a minute, and the extremities cold. Flushing with tumultuous heart action, and semi-lateral sweating. The *respiration* has been observed at 10, 15, and even 96 in a minute. Loss of power in the crico-arythenoid muscles is the common type of hysterical aphonia. The patient may be able to sing well, although unable to speak. She may speak aloud during sleep. It is well to recommend them to speak with a full chest. Tonics, rest, and full feeding are indicated. Relapses are common. There is a sense of choking, and difficulty to swallow. This has led some to observe long fasts and not take food nor liquid for as long as twenty-seven days.

Gastro-Intestinal Disorders of Hysteria.—Cases of persistent hystero-epilepsies, and of severe contractions, are not frequent in this country. The author observed five cases of hysteria lasting from fifteen to twenty-five years, all bed-ridden. Few cases died. Feed the patient, empty the bowels, give exercise, baths, tonics. It is nearly always some trick of the stomach or gastro-intestinal tract which sooner or later baffles or perplexes us. Anorexia, regurgitation, spasms on taking food by the mouth or by the rectum. Seclusion, massage, skimmed milk diet, are all useful. Absence of all possible use of body and mind. Iron in large doses, and cod-liver oil, when well borne, are useful in hysteria with anæmia. The hysteria of puberty is the most favorable. But women in good condition, fat and ruddy, with sound organs and good appetites, but ever complaining of pains and aches, and liable to a variety of hysterical phenomena, cannot usually be cured. H. D. V.

